# Synergistic MicroRNA Therapy in Liver Fibrotic Rat Using MRI‐Visible Nanocarrier Targeting Hepatic Stellate Cells

**DOI:** 10.1002/advs.201801809

**Published:** 2019-01-15

**Authors:** Jun Wu, Jinsheng Huang, Sichi Kuang, Jingbiao Chen, Xiaoxia Li, Bin Chen, Jin Wang, Du Cheng, Xintao Shuai

**Affiliations:** ^1^ The Third Affiliated Hospital Sun Yat‐sen University Guangzhou 510630 China; ^2^ PCFM Lab of Ministry of Education School of Materials Science and Engineering Sun Yat‐sen University Guangzhou 510275 China; ^3^ Department of Orthopaedics and Traumatology Nanfang Hospital Southern Medical University Guangzhou 510515 China

**Keywords:** hepatic stellate cells, liver fibrosis, miRNA delivery, magnetic resonance imaging visibility, nanomedicine, synergistic therapy

## Abstract

Liver fibrosis, as one of the leading causes of liver‐related morbidity and mortality, has no Food and Drug Administration (FDA)‐approved antifibrotic therapy yet. Although microRNA‐29b (miRNA‐29b) and microRNA‐122 (miRNA‐122) have great potential in treating liver fibrosis via regulating profibrotic genes in hepatic stellate cells (HSCs), it is still a challenge to achieve a HSC‐targeted and meanwhile noninvasively trackable delivery of miRNAs in vivo. Herein, a pH‐sensitive and vitamin A (VA)‐conjugated copolymer VA–polyethylene glycol–polyethyleneimine–poly(*N*‐(*N*′,*N*′‐diisopropylaminoethyl)‐*co*‐benzylamino) aspartamide (T‐PBP) is synthesized and assembled into superparamagnetic iron oxide (SPIO)‐decorated cationic micelle for miRNA delivery. The T‐PBP micelle efficiently transports the miRNA‐29b and miRNA‐122 to HSC in a magnetic resonance imaging‐visible manner, resulting in a synergistic antifibrosis effect via downregulating the expression of fibrosis‐related genes, including collagen type I alpha 1, α‐smooth muscle actin, and tissue inhibitor of metalloproteinase 1. Consequently, the HSC‐targeted combination therapy with miRNA‐29b and miRNA‐122 demonstrates a prominent antifibrotic efficacy in terms of improving liver function and relieving hepatic fibrosis.

## 1 Introduction

Liver fibrosis is a major liver disease characterized by accumulation of extracellular matrix (ECM) due to repeated liver injury,^1^, ^2^ and it may progress into liver cirrhosis, hepatic failure, and even hepatocellular carcinoma.^3^, ^4^ Although some antifibrotic agents, including anti‐inflammatory drugs (e.g., fresolimumab and pirfenidone), antioxidant agents (e.g., *N*‐acetylcysteine and diphenyleneiodonium), and antiviral drugs (e.g., entecavir and interferon γ), have been developed for clinical and preclinical trials, the limited efficacy and severe side effects are still main obstacles for their applications.^5^, ^6^, ^7^, ^8^, ^9^ Thus, an alternative strategy to treat hepatic fibrosis is in urgent need nowadays.

The hepatic stellate cell (HSC) is recognized as an ideal target for developing effective antifibrotic drugs due to its critical role in the initiation and progress of liver fibrosis.^2^, ^10^, ^11^, ^12^, ^13^ During liver injury, HSC is activated and then transformed to a α‐smooth muscle actin (α‐SMA)‐expressing myofibroblast‐like phenotype, which results in liver tissue contraction and generation of excessive ECM components such as collagen type I alpha 1 (COL1A1).^14^, ^15^, ^16^, ^17^ At the same time, HSC secretes tissue inhibitor of metalloproteinases (TIMPs) to inhibit the activity of metalloproteinases that are responsible for the degradation of ECM.^1^ Therefore, liver fibrosis may be reversed by suppressing the activation and proliferation of HSC, through which the ECM production can be reduced. However, most of drugs currently being used just work on the pathogenesis of liver fibrosis (e.g., hepatitis viral infection and alcohol abuse) rather than the activated HSC‐mediated liver fibrogenesis.^18^ Hence, development of novel drugs targeting HSC may be a more effective approach to treat liver fibrosis.

Nowadays, a new therapeutic technique based on microRNA (miRNA) molecules, a noncoding RNA of about 22 nucleotides, has emerged as a promising alternative to conventional drug therapies. It can regulate cell proliferation, development, and differentiation via binding to the 3′ untranslated region (3′‐UTR) of targeted mRNAs involved in initiation and progress of human diseases such as fibrotic diseases, cancer, and obesity.^19^, ^20^, ^21^, ^22^, ^23^, ^24^, ^25^ Several miRNAs, such as miRNA‐133a,^26^ miRNA‐21,^27^ miRNA‐378,^22^ miRNA‐222,^28^ miRNA‐29b,^29^ and miRNA‐122,^30^ have been reported to regulate liver fibrosis. Among these miRNAs, miRNA‐29b was a crucial therapeutic target for liver fibrosis,^31^ and miRNA‐122 was the most abundant and liver‐specific miRNA in the adult human liver.^19^, ^32^ The miRNA‐29b and miRNA‐122 inhibit collagen production of HSCs via targeting different signaling pathways of liver fibrosis, which could make HSCs more difficult to develop compensatory mechanisms escaping gene therapy. In detail, the miRNA‐29b suppressed the collagen synthesis via directly binding to the 3′‐UTR sequence of COL1A1 mRNA,^31^ indirectly blocking TGF‐β1/Smad3 or hedgehog signaling pathway,^23^, ^33^, ^34^ and upregulating PTEN signaling cascade.^35^ The miRNA‐122 inhibited the production of collagen via TGF‐β‐miR‐122‐FN1/SRF signaling cascade^36^ and the maturation of collagen via suppressing P4HA1 expression.^37^ In addition, the proliferation and activation of HSCs could be inhibited by miRNA‐29b and miRNA‐122 through blocking PI3K/Akt signaling pathway,^38^ and Bcl‐w and IGFR1 expression,^37^ respectively. Therefore, it is anticipated that a combination of miRNA‐29b and miRNA‐122 may result in a synergistic antifibrotic effect. Works about combination of two or multiple small‐molecule drugs for synergistic liver fibrosis therapy have been reported.^39^, ^40^ However, as far as we know, the combined treatment with two or more miRNAs for synergistic anti–liver fibrosis therapy has not been reported in vivo, but the miRNA‐based combination therapy has been reported to achieve synergistic outcomes in gastric cancer, glioblastoma, and cardiovascular disease.^41^, ^42^, ^43^

Due to its poor cell membrane penetration and nuclease degradation, miRNA requires a safe and highly efficient delivery in vivo. Cationic polymers such as PEI, polyamidoamine (PAMAM) dendrimers, and chitosan showed high delivery efficiency for nucleic acids.^44^, ^45^, ^46^ However, the strong positive charge and nonbiodegradability of these carriers led to cationic and accumulative toxicity. Therefore, biodegradable cationic polymers such as poly(amino acid) derivatives and low molecular weight PEI conjugates were developed to improve the biosafety of delivery carriers.^47^, ^48^, ^49^ In recent years, the design of pH‐sensitive nanocarriers for nucleic acid delivery is mainly focused on extracellular pH (pHe) and intracellular pH (pHi) responses.^50^, ^51^ The pHe‐responsive strategies, such as shielding/exposing targeting ligand and charge reversal, resolved the contradiction between blood circulation stability and targeted cell uptake. The pHi‐responsive strategies facilitated endosomal/lysosomal escape of drugs due to the “proton sponge” effect and dissociation of nanoplex. Meanwhile, targeting ligands were introduced to the surface layer of nanocarriers in order to deliver miRNA into specific cells and minimize off‐target effects.^52^, ^53^ For instance, vitamin A (VA) decoration of nanocarriers improved the cellular internalization of siRNA and drug into HSCs via an interaction between retinol‐binding protein (RBP) and RBP receptors.^54^, ^55^ On the other hand, it is important to dynamically monitor the delivery and distribution of nucleic acids in vivo. In recent years, the magnetic resonance imaging (MRI) technique has been widely applied to noninvasively track the delivery events of nanomedicines that incorporated superparamagnetic iron oxide (SPIO) nanoparticles as an MRI T_2_ contrast agent with high MRI detection sensitivity.^56^, ^57^, ^58^, ^59^

**Figure 1:**
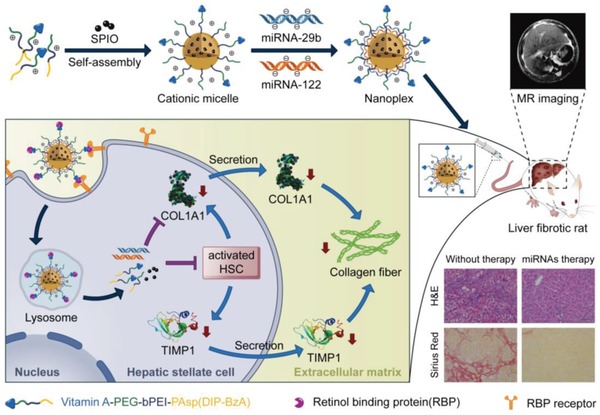
Schematic illustration of the preparation of vitamin A–decorated pH‐sensitive and SPIO‐loaded nanoplex T‐PBP@miRNA/SPIO (T‐miRNA/S) for miRNA targeting delivery, resulting in synergistically downregulated expression of liver fibrosis–related genes for alleviating liver fibrosis. The red arrows indicate the reduction of COL1A1, TIMP1, and collagen fiber. Abbreviations: COL1A1, collagen type I alpha 1 protein; TIMP1, tissue inhibitor of metalloproteinase 1; SPIO, superparamagnetic iron oxide.

In this study, an MRI‐visible cationic polymeric micelle decorated with vitamin A, VA–polyethylene glycol–polyethyleneimine–poly(*N*‐(*N*′,*N*′‐diisopropylaminoethyl)‐*co*‐benzylamino) aspartamide (VA–PEG–bPEI–PAsp(DIP–BzA))@SPIO (abbreviated as T‐PBP/S), was prepared for the HSC‐targeted delivery of miRNAs (**Figure** **1**). The miRNA‐29b and miRNA‐122 were codelivered with T‐PBP/S in animals with typical pathological characters of liver fibrosis. Serial experiments were conducted to evaluate the synergistic antifibrotic effect of miRNA‐29b and miRNA‐122, and the in vivo distribution of miRNA during treatment was monitored with MRI.

## 2 Results and Discussion

### 2.1 Preparation and Characterization of Nanoplexes

**Figure 2:**
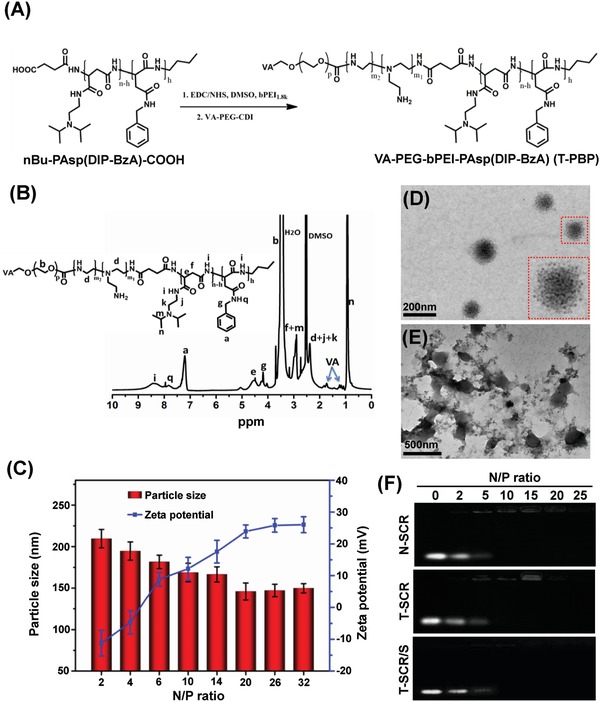
Synthesis and characterization of the polymer and nanoplexes. A) Synthetic route of the triblock copolymer VA–PEG–bPEI–PAsp(DIP–BzA) denoted as T‐PBP. B) ^1^H NMR spectrum of the T‐PBP polymer. C) Particle size and zeta potential of SPIO‐loaded nanoplex (T‐SCR/S) with different N/P ratios at pH 7.4. Data are shown as mean ± SD, *n* = 3. TEM images of T‐SCR/S (N/P 10) at D) pH 7.4 and E) pH 5.0. F) Electrophoretic mobility of SCR in agarose gel after complexation with SPIO‐free micelle (N‐SCR and T‐SCR) and SPIO‐loaded micelle (T‐SCR/S) at various N/P ratios. Abbreviations: SCR, scrambled miRNA; N‐SCR, nontargeting PBP micelle complexing SCR; T‐SCR, targeting T‐PBP micelle complexing SCR; T‐SCR/S, T‐PBP micelle complexing SCR and encapsulating SPIO.

The VA‐terminated pH‐sensitive copolymer, VA–PEG–bPEI–PAsp(DIP–BzA) or T‐PBP, was synthesized according to our recent report (**Figure** **2**A; Figure S1, Supporting Information)^60^ and characterized by gel permeation chromatography (GPC) and ^1^H NMR. GPC chromatograms showed a unimodal molecular weight distribution for all copolymers and an increase in the molecular weight of final copolymer compared with the prepolymer, suggesting that VA‐terminated PEG was conjugated to final copolymer (Figure S2 and Table S2, Supporting Information). The chemical shifts in the ^1^H NMR spectrum of final copolymer at 3.6, 7.25, and 0.9 ppm were ascribed to —OC***H***_2_C***H***_2_O— of PEG, benzyl group of BzA, and isopropyl group of DIP, respectively (Figure 2B). In particular, the characteristic chemical shifts of vitamin A were at 1.05–1.35 and 1.75–1.92 ppm, which confirmed that VA‐terminated final copolymer was successfully synthesized. The nontargeting copolymer, polyethylene glycol–polyethyleneimine–poly(*N*‐(*N*′,*N*′‐diisopropylaminoethyl)‐*co*‐benzylamino)aspartamide abbreviated as PEG–bPEI–PAsp(DIP–BzA) or PBP, was also synthesized using the same procedure as the VA‐terminated copolymer.

Then, the T‐PBP and PBP copolymers were self‐assembled into SPIO‐free and SPIO‐encapsulated cationic micelles, which were named T‐PBP (blank T‐PBP micelle), T‐PBP/S (SPIO‐encapsulated T‐PBP micelle), PBP (blank PBP micelle), and PBP/S (SPIO‐encapsulated PBP micelle). Furthermore, nanoplexes were prepared by using the above cationic micelles to complex miRNA including miRNA‐29b, miRNA‐122, and scrambled miRNA (SCR). The SCR‐complexed nanoplexes were named T‐SCR (T‐PBP micelle complexing SCR), T‐SCR/S (T‐PBP micelle complexing SCR and encapsulating SPIO), N‐SCR (PBP micelle complexing SCR), and N‐SCR/S (PBP micelle complexing SCR and encapsulating SPIO).

As shown in Figure 2C, the particle size of T‐SCR/S nanoplex decreased along with an increase in N/P ratio, calculated as the molar number of nitrogen atoms in the PEI block over that of the phosphate groups in the miRNA, and leveled off at ≈149.8 ± 5.6 nm (N/P ≥ 20) as measured by dynamic light scattering (DLS). Moreover, zeta potential of the nanoplex increased along with an increase in N/P ratio because more amino groups were present on the surface of nanoplex at higher N/P ratio. It has been reported that nanoplex showing weak positive charge and small particle size may well protect nucleic acids from the degradation of nucleases and meanwhile effectively deliver nucleic acids into cells.^47^, ^48^ Thus, the nanoplex of N/P 10 with moderate positive charge (+12.2 ± 3.6 mV) and relatively small particle size (168.8 ± 9.2 nm) and particle distribution index (PDI = 0.11) was chosen for the biological experiments (Table S3, Supporting Information).

The morphology of nanoplex was revealed by transmission electron microscopy (TEM) analysis. At pH 7.4, the nanoplex of N/P 10 showed roughly spherical shape and fairly uniform size of about 155.0 ± 10.8 nm in diameter (Figure 2D), which was slightly smaller than that detected by DLS measurement. On the contrary, random polymeric aggregates were observed at pH 5.0 (Figure 2E), which had a particle size of 1088 ± 108.5 nm as detected by the DLS measurement (Table S3, Supporting Information), likely because the complete protonation of DIP caused a hydrophilic transition of the PAsp(DIP–BzA) block, which induced the disassembly of nanoplex.^60^ As shown in Figure S3 (Supporting Information), the fluorescent intensity of Cy3‐labeled miRNA increased via pH 5.0 preincubation as compared with pH 7.4, which indicated that Cy3‐labeled miRNA was released from T‐SCR/S nanoplex due to the disassembly of T‐SCR nanoplex at pH 5.0 lowering miRNA complexation ability of free VA–PEG–bPEI–PAsp(DIP–BzA) during endo‐lysosomal escape.^61^ To further elucidate the potential impact of pH‐sensitive DIP groups on miRNA release, the control nanoplex (C‐SCR/S) was prepared at N/P 10 by using SPIO‐loaded cationic micelle of VA–PEG–bPEI–PAsp(BzA) without DIP grafting to complex SCR. The solutions of Cy3‐labeled C‐SCR/S showed similar fluorescent intensities no matter whether preincubated at pH 5.0 or not (Figure S3A, Supporting Information). As shown in Figure S3B (Supporting Information), the tailing bands of miRNA and different migration distances reflected the incomplete complexation of miRNA with T‐SCR/S nanoplex, but miRNA migration in gel electrophoresis was not strengthened due to preincubation of C‐SCR/S at pH 5.0. These results indicated that introduction of the pH‐sensitive DIP groups may enhance pH‐sensitive miRNA release of nanoplex, likely due to low pH‐inducible disassembly of micelle core, which lowers the nanoplex stability and capacity to condense miRNA. Hence, the proton sponge effect of PEI and pH‐sensitive feature of DIP moiety both may facilitate lysosomal escape and cytoplasm release of miRNA. If PAsp(DIP–BzA) was replaced with poly(lactic‐*co*‐glycolic acid), the aggregates were not expected to collapse quickly at low pH, which was not beneficial to intracellular miRNA release.

### 2.2 miRNA Complexation Ability of Nanocarrier

Agarose gel electrophoresis was further performed to assess the miRNA complexation ability of nanoplexes (N‐SCR, T‐SCR, and T‐SCR/S). As shown in Figure 2F, free miRNA with negative charge migrated to the anode under electric field and showed a sharp fluorescent band after staining with ethidium bromide (EB). However, the miRNA migration in agarose gel was retarded due to the formation of nanoplexes between cationic micelles and miRNA at various N/P ratios. At N/P ratios below 10, the miRNA migration was partly retarded because an insufficient amount of cationic micelle was unable to completely neutralize the negatively charged miRNA. However, at N/P ratios above 10, the band of migrated miRNA disappeared completely, indicating a complete miRNA complexation. Furthermore, the electrophoretic behaviors of miRNA complexed with the T‐PBP micelle (T‐SCR) and SPIO‐encapsulated T‐PBP micelle (T‐SCR/S) seemed very similar to that of miRNA complexed with the PBP micelle (N‐SCR). These results demonstrated that the cationic micelles could effectively complex miRNA, and the vitamin A decoration and SPIO encapsulation had negligible effect on the miRNA complexation of cationic micelles.

### 2.3 Optimization of Complexation between miRNA and Nanocarrier

As shown in Figure S4 (Supporting Information), T‐SCR/S and N‐SCR/S nanoplexes remained stable in the Dulbecco's modified Eagle medium (DMEM, pH 7.4). Moreover, the particle size of these two nanoplexes only showed a slight increase and then remained stable over 48 h in serum‐containing DMEM. 3‐(4,5‐Dimethylthiazol‐2‐yl)‐2,5‐diphenyltetrazolium bromide (MTT) assay was performed to evaluate the cationic cytotoxicity of various nanoplexes in HSCs, using the nontransfected cells as a control. Even at a high polymer concentration of 400 µg mL^−1^, the cell viabilities in all groups were still above 88.5% (Figure S5A, Supporting Information), indicating that the SCR‐complexed nanoplexes had little cytotoxicities. In addition, the cytotoxicities of nontargeting nanoplex (N‐SCR) and targeting nanoplex (T‐SCR) at various N/P ratios were compared. Below N/P 10, both nontargeting and targeting nanoplexes showed negligible cytotoxicities (Figure S5B, Supporting Information). Above N/P 10, the targeting nanoplex showed higher cytotoxicity than the nontargeting one, which might be due to the higher transfection efficiency of the targeting one. The N/P ratio–dependent transfection efficiency was also quantified using flow cytometry assay. As shown in Figure S5C,D (Supporting Information), the highest transfection efficiency was observed in the cells receiving T‐SCR nanoplex (N/P 10), i.e., 97.5% of Cy3‐positive cells, indicating more internalization of T‐SCR into cells. In addition to the cell uptake level, the cytotoxicity of a nanoplex was also decided by its surface charge, and highly positive nanoparticles were reported to be more toxic.^45^, ^52^ Therefore, T‐SCR nanoplexes above N/P 10 possessed higher positive charge and thus even higher cytotoxicity. Assumably, incubation with highly cytotoxic nanoplexes may also affect the cell state, thus leading to even low cell uptake of nanoplexes with too high N/P ratios (e.g., above 10). These results suggested that N/P 10 was indeed an ideal condition to prepare nanoplex for miRNA delivery.

### 2.4 MRI‐Visible HSC‐Targeted Delivery of miRNA

**Figure 3:**
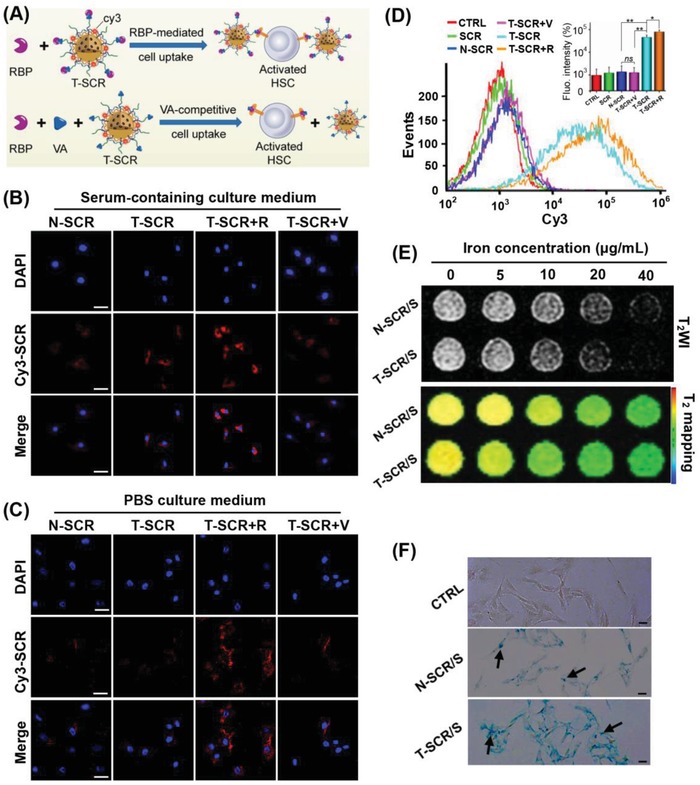
HSC‐targeted miRNA delivery and MRI capacity of the targeting nanoplexes. A) Schematic illustration of the RBP‐mediated cell uptake and competitive inhibition effect of excessive free vitamin A. CLSM images of HSCs after incubation with N‐SCR, T‐SCR, T‐SCR+R, and T‐SCR+V in B) serum‐containing culture medium for 2 h and C) PBS culture medium for 0.5 h. The blue and red fluorescence indicate the DAPI‐labeled nuclei and Cy3‐labeled SCR, respectively. Scale bars represent 20 µm. D) Quantification of Cy3‐labeled miRNA transfection efficiency mediated by naked SCR and various nanoplexes (i.e., N‐SCR, T‐SCR, T‐SCR+R, and T‐SCR+V) for 2 h in serum‐containing culture medium. Data are shown as mean ± SD, *n* = 3. *P < 0.05, **P < 0.01, and ns represents no significant difference. E) T_2_‐weighted imaging (T_2_WI) and T_2_ mapping imaging of HSCs after incubation with N‐SCR/S and T‐SCR/S at various Fe concentrations. F) Intracellular distribution of SPIO using Prussian blue staining. Cells were incubated with SPIO‐encapsulated nanoplexes for 2 h. Scale bars represent 100 µm. The black arrows mark the SPIO in HSCs. All nanoplexes were prepared at an N/P ratio of 10. Abbreviations: RBP, retinol‐binding protein; CTRL, cells without treatment; N‐SCR, PBP micelle complexing SCR; T‐SCR, T‐PBP micelle complexing SCR; T‐SCR+V, T‐SCR nanoplex plus preincubation with excessive vitamin A; T‐SCR+R, T‐SCR nanoplex plus preincubation with RBP at a concentration of 0.7 µg mL^−1^; N‐SCR/S, SPIO‐encapsulated N‐SCR; T‐SCR/S, SPIO‐encapsulated T‐SCR.

The complex of VA and RBP could specifically bind to the RBP receptor overexpressed on the cytomembrane of activated HSCs (**Figure** **3**A).^54^ Thus, the HSC‐targeted miRNA delivery may be achieved by introducing VA ligand on the surface of PBP micelle. To observe the cell uptake, SCR and nuclei of HSCs were labeled with Cy3 (red fluorescence) and 4′,6‐diamidino‐2‐phenylindole (DAPI, blue fluorescence), respectively. As shown in Figure 3B, the cells transfected with Cy3‐labeled T‐SCR in serum‐containing medium showed stronger red fluorescence than those transfected with Cy3‐labeled N‐SCR, likely due to RBP present in the serum. The addition of extra RBP (T‐SCR+R group) further increased the fluorescence intensity of cells receiving Cy3‐labeled T‐SCR. In contrast, the red fluorescence of HSCs preincubated with free vitamin A (T‐SCR+V group) was much weaker than that of HSCs just receiving Cy3‐labeled T‐SCR, which was attributed to the competitive inhibition effect of excessive vitamin A. To further elucidate the mechanism of VA–RBP complex–mediated HSC‐targeting delivery, the serum‐containing medium was replaced with phosphate‐buffered saline (PBS) solution. Considering that long incubation time was harmful to cells if the PBS was applied, the shorter incubation time (0.5 h) was used in this case. As shown in Figure 3C, the cells transfected with Cy3‐labeled T‐SCR in the RBP‐absent PBS showed almost the same level of red fluorescence intensity as those transfected with N‐SCR, but the addition of RBP remarkably increased the red fluorescence intensity of cells receiving Cy3‐labeled T‐SCR. These results clearly indicated that the VA‐enhanced transfection efficiency was mediated by the VA–RBP complex. Notably, upon the preincubation with excessive free VA in the PBS solution, the red fluorescence intensity of cells receiving Cy3‐labeled T‐SCR was decreased to the similar level of those receiving Cy3‐labeled N‐SCR, which verified the competitive inhibition effect of free VA once again.

Moreover, the miRNA delivery efficiencies of various nanoplexes in RBP‐containing medium were quantified by flow cytometry (Figure 3D). Cells incubated with targeting nanoplex (T‐SCR) showed much higher fluorescence intensity than those incubated with N‐SCR (4.6 × 10^4^ vs 1.4 × 10^3^ a.u.). The addition of extra RBP to the culture medium (T‐SCR+R group) further increased the fluorescence intensity of T‐SCR‐incubated HSCs by 0.7 times. However, the fluorescence intensity of T‐SCR‐incubated cells was significantly reduced upon preincubation with excessive vitamin A (1.4 × 10^3^ vs 4.6 × 10^4^ a.u.). These results demonstrated that the internalization of T‐SCR into HSCs was mediated by a ligand–receptor interaction. In other words, as the vitamin A transporter, RBP binding to the vitamin A–decorated nanoplex enhanced the specific interaction with the overexpressed RBP receptors on HSCs for efficient cell uptake.^54^, ^55^ As RBP binding free vitamin A lost ability to bind T‐SCR, the cell uptake of nanoplex was blocked remarkably.

Admittedly, a nanocarrier should also possess an ability of lysosomal escape to prevent miRNA degradation inside lysosomes.^45^ Thus, the lysosomes of HSC were labeled with LysoTracker (green fluorescence) to show the lysosomal escape of miRNA (Figure S6, Supporting Information). The fluorescence of Cy3‐SCR (red) and LysoTracker (green) overlapped to show yellow stains at 0.5 h after transfection, indicating the location of SCR inside lysosome. However, the fluorescence of the Cy3‐SCR was separated from that of lysosome at 2 h after transfection, indicating lysosome escape of miRNAs likely due to the proton sponge effect of PEI and DIP groups.^46^, ^60^

**Figure 4:**
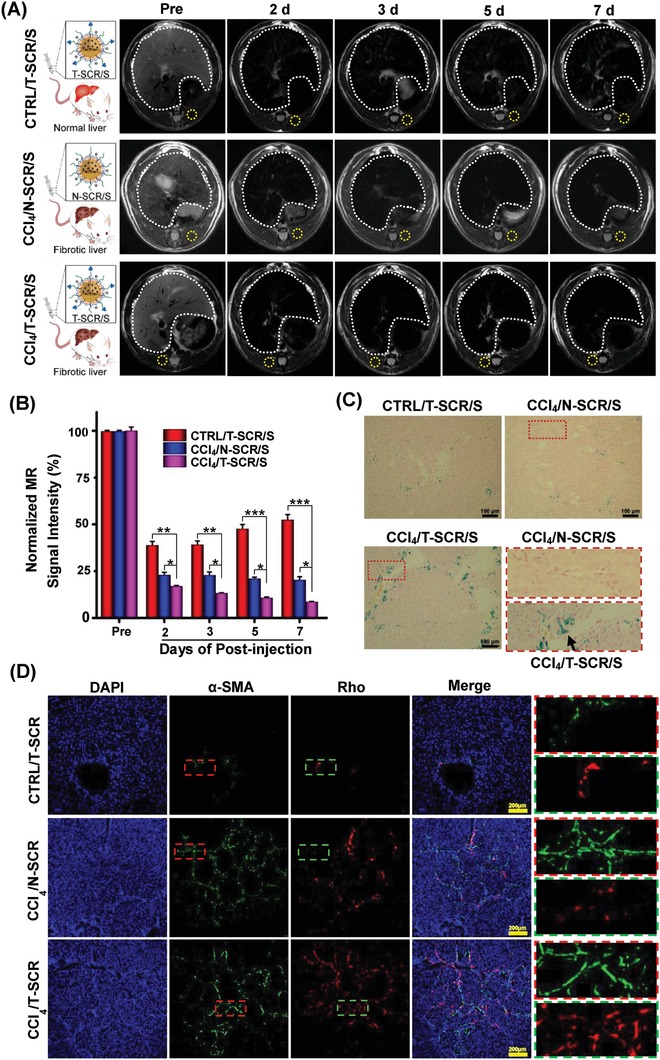
In vivo distribution of the nontargeting and targeting nanoplexes in normal rats and liver fibrotic rats using MRI and ex vivo fluorescence imaging. A) T_2_‐weighted imaging of liver in rats before (Pre) and after i.v. injection of N‐SCR/S and T‐SCR/S at various time points. SPIO dose: 10 mg Fe kg^−1^ body weight. The white dotted portions indicate liver tissue, and the yellow dotted portions indicate the muscle tissue. B) T_2_‐weighted MR signal intensity of liver normalized by referring to that of the muscle tissue. Data are shown as mean ± SD, *n* = 3. *P < 0.05, **P < 0.01, and ***P < 0.001. C) Prussian blue staining of liver sections excised from rats receiving N‐SCR/S and T‐SCR/S. The areas marked with dotted red rectangle are amplified to clearly show the SPIO nanoparticles. The black arrows mark the SPIO. D) Colocalization of nanoplexes and HSCs in vivo. Blue, red, and green fluorescence indicate the DAPI‐labeled nuclei, Rho‐labeled nanocarrier, and AF488‐labeled antibody against α‐SMA (a biomarker of HSCs), respectively. Scale bars represent 100 and 200 µm in parts (C) and (D), respectively. Abbreviations: Rho, rhodamine B; CTRL/T‐SCR/S, normal rats receiving T‐SCR/S; CCl_4_/N‐SCR/S, CCl_4_‐induced liver fibrotic rats receiving N‐SCR/S; CCl_4_/T‐SCR/S, CCl_4_‐induced liver fibrotic rats receiving T‐SCR/S; CTRL/T‐SCR, normal rats receiving Rho‐labeled T‐SCR; CCl_4_/N‐SCR, CCl_4_‐induced liver fibrotic rats receiving Rho‐labeled N‐SCR; CCl_4_/T‐SCR, CCl_4_‐induced liver fibrotic rats receiving Rho‐labeled T‐SCR.

Incorporation of SPIO into the nanoplexes enabled MRI monitoring of miRNA delivery. The T_2_ relaxivities (*r*_2_) of nanoplexes T‐SCR/S (T‐PBP micelle encapsulating SCR and SPIO) and N‐SCR/S (PBP micelle encapsulating SCR and SPIO) reached 110.6 and 107.2 Fe mM^−1^ s^−1^, respectively, which were significantly higher than that of WSPIO (*r*_2_ = 35.9 Fe mM^−1^ s^−1^) and suggested a high MRI T_2_ sensitivity of the SPIO‐labeled nanoplexes (Figure S7, Supporting Information). As shown in Figure 3E, MRI could monitor the internalization of nanoplexes into HSCs. The targeting nanoplex entered the HSCs more efficiently, resulting in more obvious decrease in the T_2_‐weighted signals of MRI. In addition, cells incubated with T‐SCR/S showed much more Prussian blue stains than those incubated with N‐SCR/S, which demonstrated that the introduction of vitamin A could effectively enhance miRNA delivery into HSCs (Figure 3F). Then, the liver fibrotic rats receiving N‐SCR/S or T‐SCR/S and normal rats receiving T‐SCR/S were subjected to in vivo MRI scanning. At 2 d after intravenous (i.v.) injection, the liver fibrotic rats receiving T‐SCR/S showed 60.0% reduction compared with the normal rats receiving T‐SCR/S and 33.3% reduction compared with the liver fibrotic rats receiving N‐SCR/S in the liver T_2_‐weighted signal intensity (**Figure** **4**A,B). These results indicated an enhanced liver accumulation of T‐SCR/S in liver fibrotic rats, which was reasonable because the activated HSCs therein allowed a more effective retention of targeting nanoplex in fibrotic liver.^14^, ^54^ Compared with the normal liver of CTRL/T‐SCR group, the fibrotic liver of CCl_4_/N‐SCR possessed more activated Kupffer cells that phagocytosed N‐SCR/S as a foreign substance,^62^ resulting in lower T_2_‐weighted MR signal intensity of fibrotic liver. From 2 to 7 d after i.v. administration, normal rats receiving T‐SCR/S showed gradual recovery to 52.4% from 38.8% in the liver T_2_‐weighted signal intensity, whereas the liver fibrotic rats receiving N‐SCR/S exhibited steady liver T_2_‐weighted signal intensity. In contrast, the liver fibrotic rats receiving T‐SCR/S showed a persistent decrease in the liver T_2_‐weighted signal intensity, i.e., from 16.9% at 2 d to 8.6% at 7 d after i.v. injection (Figure 4B). These results indicated that the HSC‐targeted nanoplex T‐SCR/S offered a great potential to monitor miRNA delivery in vivo and possibly to detect activated HSCs in liver fibrosis. Furthermore, the Prussian blue staining on liver tissue sections further verified the accumulation of SPIO in liver. As shown in Figure 4C, the liver tissue sections from animals receiving T‐SCR/S showed much more SPIO accumulation (blue stains), which was in line with the results of MRI measurements in vivo.

The miRNA distribution in liver tissues was further investigated via fluorescent imaging of rhodamine B (Rho)‐labeled T‐SCR (red fluorescence) and Alexa Fluor 488 (AF488)‐stained α‐SMA (green fluorescence) as a biomarker of HSCs. Liver tissue sections from the liver fibrotic animals showed much stronger green fluorescence (α‐SMA) than those from the normal animals, and sections from the liver fibrotic animals receiving T‐SCR/Rho showed the highest intensity of red fluorescence (Figure 4D). Moreover, the colocalization of α‐SMA and Rho to some extent evidenced that Rho‐labeled miRNA distribution was delivered into the activated HSCs expressing α‐SMA in fibrotic liver.

Apparently, although cationic polymers such as PEI with/without PEG modification have been used to deliver nucleic acids for years, the delivery of nucleic acids such as miRNA still faces challenges. First, high molecular weight PEI (e.g., 25 kDa PEI) is generally reported to effectively complex nucleic acids, whereas nucleic acid decomplexation with high molecular weight PEI inside cells is difficult solely via the proton sponge effect of PEI.^50^ Here, we demonstrated that the introduction of pH‐sensitive DIP groups not only allowed effective miRNA complexation with small molecular weight PEI but also promoted intracellular miRNA release (see Figure S3 in the Supporting Information). Second, although vitamin A has been used as a targeting ligand for HSC‐targeted siRNA delivery,^54^ the strategy has not been applied in miRNA therapy yet. Third, the miRNA therapy integrating pH‐sensitive miRNA release, HSC‐targeted miRNA delivery, and MRI monitoring has not been reported so far.

### 2.5 Level and Retention of miRNA‐29b and miRNA‐122 in Fibrotic Liver

**Figure 5:**
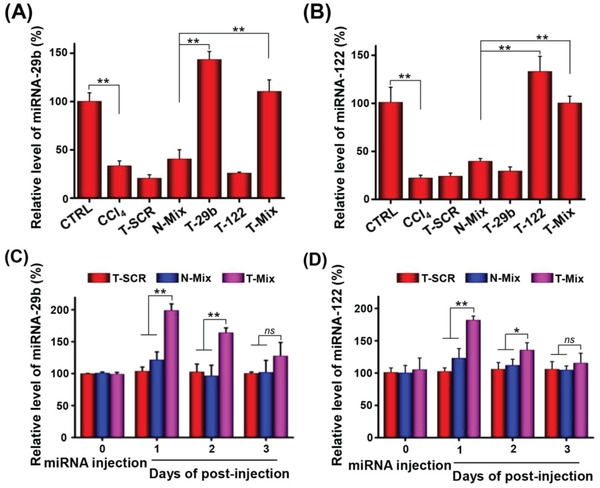
Content and time duration of the miRNA in CCl_4_‐induced liver fibrotic rats with/without extrinsic miRNA administration as determined by RT‐PCR. Relative levels of A) miRNA‐29b and B) miRNA‐122 in livers at 24 h after i.v. administration of different nanoplexes. Content of C) miRNA‐29b and D) miRNA‐122 at 1, 2, and 3 d after i.v. injection of different nanoplexes. The dose of miRNA‐29b and miRNA‐122 was 1 mg kg^−1^ body weight. Data are shown as mean ± SD, n = 3. *P < 0.05 and **P < 0.01. Abbreviations: N‐Mix, PBP micelle complexing miRNA‐29b and miRNA‐122; T‐Mix, T‐PBP micelle complexing miRNA‐29b and miRNA‐122.

The miRNA‐29b and miRNA‐122 were reported to reduce ECM accumulation by downregulating several fibrosis‐related proteins involved in HSC activation and metabolism of collagen.^31^, ^34^, ^36^, ^37^, ^38^ Therefore, codelivery of these two miRNAs to HSCs may be an effective approach for treating liver fibrosis. To optimize the regimen of miRNA therapy, the level and retention time of miRNAs in liver fibrotic rats were investigated. As shown in **Figure** **5**A,B, the fibrotic liver of rats significantly decreased miRNA‐29b and miRNA‐122 levels at 6 weeks after CCl_4_ treatment to induce fibrosis, as compared to the normal rats (CTRL, control group). Therefore, delivering miRNA‐29b and miRNA‐122 to HSCs is meaningful for alleviating liver fibrosis. In our study, administration of targeting nanoplexes T‐29b carrying miRNA‐29b, T‐122 carrying miRNA‐122, and T‐Mix carrying a mixture of miRNA‐29b/miRNA‐122 increased the levels of miRNA‐29b and miRNA‐122 in fibrotic liver (Figure 5A,B). In contrast, the T‐SCR treatment failed to increase the levels of these two miRNAs. Next, the retention of miRNA‐29b and miRNA‐122 was evaluated in liver fibrotic rats at 1, 2, and 3 d after administration of nanoplexes carrying miRNA. As shown in Figure 5C,D, the miRNA‐29b and miRNA‐122 levels in rats receiving T‐Mix increased at 1 d and then gradually decreased to the levels in rats receiving T‐SCR at 3 d after administration. Therefore, nanoplexes were administered twice a week in the treatment of liver fibrotic rats. In addition, there was no significant difference in the levels of miRNA‐29b and miRNA‐122 between rats receiving T‐SCR and rats receiving N‐Mix. These results strongly evidenced that the HSC‐targeted codelivery of miRNA‐122 and miRNA‐29b played the most important role in the antifibrotic therapy.

### 2.6 Synergistic Inhibition of Profibrotic Gene Expression

**Figure 6:**
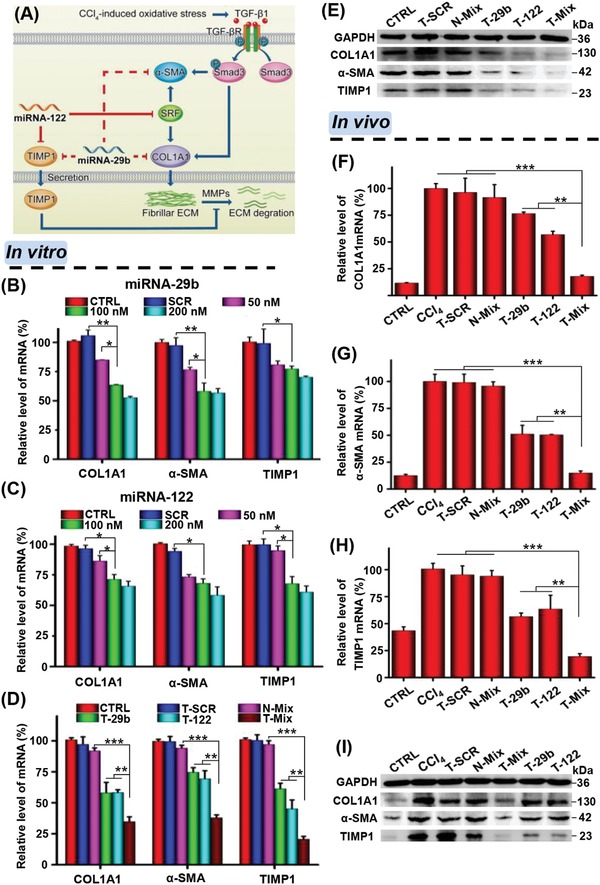
miRNA dose and synergistic effect of miRNA‐29b and miRNA‐122 on inhibition of fibrosis‐related gene expressions in vitro and in vivo. A) Schematic illustration of the **s**ynergistic antifibrosis mechanism of combined therapy with miRNA‐29b and miRNA‐122. Relative mRNA levels of COL1A1, α‐SMA, and TIMP1 in HSCs incubated with B) miRNA‐29b and C) miRNA‐122 at 0, 50, 100, and 200 × 10^−9^ m complexed with T‐PBP micelle. Synergistic effect of a combination of miRNA‐29b and miRNA‐122 on D) mRNA and E) protein expressions of COL1A1, α‐SMA, and TIMP1 as evaluated by real‐time PCR and Western blot. The concentration of miRNA was 100 × 10^−9^ m, and 50 × 10^−9^ m miRNA‐29b and 50 × 10^−9^ m miRNA‐122 were used for N‐Mix and T‐Mix groups, respectively. F–H) Relative mRNA and I) protein expressions of COL1A1, α‐SMA, and TIMP1 in the liver sliced from rats receiving different treatments. Data are shown as mean ± SD, *n* = 3. *P < 0.05, **P < 0.01, and ***P < 0.001. Abbreviations: CTRL in parts (B)–(E), cells without treatment; T‐SCR, T‐PBP micelle complexing SCR; N‐Mix, PBP micelle complexing miRNA‐29b and miRNA‐122; T‐29b, T‐PBP micelle complexing miRNA‐29b; T‐122, T‐PBP micelle complexing miRNA‐122; T‐Mix, T‐PBP micelle complexing miRNA‐29b and miRNA‐122; CTRL in parts (F)–(I), normal rat treated with equal quantity of olive oil; CCl_4_, CCl_4_‐induced liver fibrotic rat just treated with PBS. Rats, except in the CTRL group, were pretreated with CCl_4_ to induce liver fibrosis.

As shown in **Figure** **6**A, the miRNA‐29b and miRNA‐122 can act on different regulation sites of liver fibrosis–related gene network, which might synergistically reduce accumulation of collagen in the injured liver.^31^, ^33^, ^34^, ^36^, ^37^, ^38^ Therefore, the enhanced antifibrotic effect of a combination therapy of miRNA‐29b and miRNA‐122 may result from the synergistic regulation effect on fibrosis‐related genes such as COL1A1, α‐SMA, and TIMP1. First, we evaluated the miRNA dose‐dependent inhibition effect on cell proliferation and profibrotic gene expression. As shown in Figure S8 (Supporting Information), targeting codelivery of miRNA‐29b and miRNA‐122 resulted in the greatest inhibition of HSC proliferation at all miRNA concentrations. Moreover, both the miRNA‐29b and miRNA‐122 treatments could remarkably inhibit the expression of profibrotic genes such as COL1A1, α‐SMA, and TIMP1 at 100 × 10^−9^ m miRNA (Figure 6B,C). At even higher miRNA concentration of 200 × 10^−9^ m, such inhibition effect was not further enhanced. Using the nanoplexes carrying single miRNA, the concentration of 100 × 10^−9^ m was found to be the optimized one for both miRNA‐29b and miRNA‐122. Therefore, the 1:1 complex was prepared, and two miRNAs were applied at the same concentration of 100 × 10^−9^ m. As shown in Figure 6D, a combination therapy of miRNA‐29b and miRNA‐122 (T‐Mix) resulted in an obviously higher inhibition efficiency on gene expressions of COL1A1 (65.2 ± 3.9% vs 42.0 ± 8.5% of T‐29b or 41.7 ± 2.3% of T‐122), α‐SMA (62.1 ± 2.3% vs 25.4 ± 3.7% of T‐29b or 30.6 ± 6.5% of T‐122), and TIMP1 (79.4 ± 2.2% vs 38.8 ± 4.5% of T‐29b or 54.9 ± 7.1% of T‐122) than the single miRNA therapy, i.e., T‐29b or T‐122. The nontargeting combination therapy of miRNA‐29b and miRNA‐122 (N‐Mix) appeared much less effective. In addition, the gene regulation abilities of combined treatment of two miRNAs were also evaluated at protein level, showing that the T‐Mix treatment resulted in the least protein expressions of COL1A1, α‐SMA, and TIMP1 in HSCs (Figure 6E).

The codelivery of miRNA‐29b and miRNA‐122 to regulate the liver fibrosis–related gene expression in vivo was further investigated. Previous studies have demonstrated that the synergistic therapeutic effect was enhanced when two drugs were loaded in one nanocarrier and codelivered, because the codelivery strategy allowed two drugs to be delivered into the same cell at well‐controllable ratios.^63^, ^64^ Thus, the codelivery of miRNA‐29b and miRNA‐122 may achieve better antifibrotic effect than simply mixing miRNA‐29b nanoplex and miRNA‐122 nanoplex together and injecting. As shown in Figure 6F–H, the fibrotic liver of rats with CCl_4_ treatment showed obviously upregulated expressions of COL1A1, TIMP1, and α‐SMA genes as compared with normal rats (CTRL group), which indicated the successful establishment of liver fibrotic model. The SCR treatment (T‐SCR) showed no inhibitory effect on target gene expressions, and the nontargeting miRNA treatment (N‐Mix) only slightly inhibited target gene expressions. In contrast, both the T‐29b treatment and T‐122 treatment resulted in significant inhibitions of COL1A1, α‐SMA, and TIMP1 gene expressions. Excitingly, the T‐Mix treatment further lowered the expressions of these liver fibrosis–related genes. In addition, analyses of target gene expressions at protein levels obtained consistent results as well (Figure 6I).

The combination therapy of miRNA‐29b and miRNA‐122 indeed led to much better suppression of HSC activation and proliferation than the therapy using miRNA‐29b or miRNA‐122 alone. The protein level of α‐SMA, a typical biomarker of activated HSC,^17^, ^65^ was lowered remarkably in fibrotic liver of animals receiving combined treatment than the single miRNA treatment. These results suggested that the combination treatment of miRNA‐29b and miRNA‐122 could synergistically reduce liver fibrosis via targeting multiple regulation sites involved in synthesis and degradation of collagen.

### 2.7 Liver Fibrosis Reduced by miRNA‐29b and miRNA‐122 Treatment In Vivo

**Figure 7:**
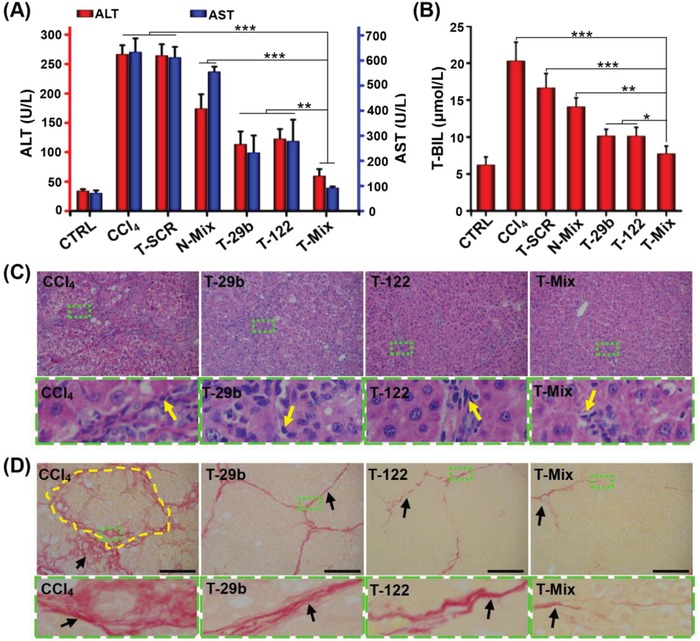
In vivo synergistic antifibrotic effect of miRNA‐29b and miRNA‐122. Effect of various miRNA treatments on A) serum ALT and AST levels and B) T‐BIL levels. Data are shown as mean ± SD, *n* = 3. *P < 0.05, **P < 0.01, and ***P < 0.001. C) H&E and D) Sirius red staining of liver specimen of CCl_4_‐induced rats after i.v. administration of various nanoplexes. The areas marked with dotted green rectangle are enlarged to reveal the changes of pathological structure. The yellow arrows indicate the inflammatory cells, and the yellow dotted portion indicates the pseudolobule in liver. The black arrows mark the collagen fiber. Scale bars represent 100 µm.

The functional and histopathological changes of liver tissue were evaluated to explore whether the miRNA therapy could reduce liver fibrosis and recover liver functions. The levels of alanine transaminase (ALT), aspartate transaminase (AST), and total bilirubin (T‐BIL) in serum were critical parameters to assess liver function damage.^66^ Compared with the CTRL group (normal rats), the ALT, AST, and T‐BIL levels in liver fibrotic rats all increased significantly (**Figure** **7**A,B). The serum ALT, AST, and T‐BIL levels were not decreased via T‐SCR treatment. In contrast, the T‐29b, T‐122, and T‐Mix treatments significantly lowered the serum levels of ALT, AST, and T‐BIL. Although the T‐Mix treatment showed slightly higher levels of the serum ALT, AST, and T‐BIL than the CTRL group, the T‐Mix treatment significantly decreased the levels of the serum ALT, AST, and T‐BIL as compared with the CCl_4_ group, which indicated that the combined miRNA therapy significantly improved liver function and alleviated liver fibrosis. To evaluate the histopathological changes, liver tissue sections were subjected to hematoxylin and eosin (H&E) and Sirius red staining. As shown in Figure 7C,D and Figure S9 (Supporting Information), the liver tissues of normal animals showed no inflammation, hepatic lobe reconstruction, and collagen deposition. In contrast, neutrophil infiltration and massive collagen deposition were clearly seen in liver tissues of fibrotic animals treated with PBS and T‐SCR. Although the treatment using T‐29b or T‐122 alone reduced the collagen fibers in fibrotic liver, the combined treatment with T‐Mix (nanoplex targeting HSC) resulted in the least collagen accumulation.

## 3 Conclusion

A vitamin A–terminated copolymer VA–PEG–bPEI–PAsp(DIP–BzA) was synthesized and self‐assembled to cationic nanoplex (T‐PBP). MRI contrast agent SPIO was encapsulated into the core of T‐PBP, and miRNAs were effectively complexed in the cationic bPEI interlayer. The vitamin A conjugation endowed the nanoplex with HSC‐targeting function, which appeared essential for realizing the synergistic effect of miRNA‐29b and miRNA‐122. In animal study, the HSC‐targeted combination treatment of miRNA‐29b and miRNA‐122 showed significant inhibitory effect on fibrosis‐related gene expressions, whereas the nontargeting combination treatment showed almost no effect. Consequently, the liver fibrosis was remarkably alleviated, and the liver functions were obviously recovered. This study showed the great potential of MRI‐visible nanocarrier targeting hepatic stellate cells to mediate highly effective anti–liver fibrosis treatment of miRNAs.

## 4 Experimental Section

*Materials*: VA, RBP, MTT, α‐methoxy‐ε‐hydroxy‐poly(ethylene glycol) (*M*_n_ = 2 kDa), hyperbranched PEI (*M*_n_ = 1.8 kDa), *N*,*N*′‐carbonyldiimidazole, *n*‐butylamine, succinic anhydride, *N*,*N*‐diisopropylaminoethylamine, benzylamine, dicyclohexylcarbodiimide, and DAPI were purchased from Sigma‐Aldrich (St. Louis, USA). SPIO nanoparticles were synthesized using the solvothermal method.^67^

An immortalized rat HSC cell line (HSC‐T6) from the Institute of Biochemistry and Cell Biology, the Chinese Academy of Sciences (Shanghai, China), was used for in vitro experiments. DMEM, PBS, fetal bovine serum (FBS), and penicillin–streptomycin were purchased from Gibco BRL (Carlsbad, USA). Cy3‐labled miRNA, SCR, miRNA‐29b (sense, 5′‐UUUCAUAUGGUGGUUUAGAUUU‐3′; antisense, 5′‐AUCUAAACCACCAUAUGAAAUU‐3′), and miRNA‐122 (sense, 5′‐UGGAGUGUGACAAUGGUGUUUG‐3′; antisense, 5′‐AACACCAUUGUCACACUCCAUU‐3′) were purchased from Genpharm (Suzhou, China). PrimeScript RT Master Mix and FastStart Universal SYBR Green Master (ROX) were purchased from Takara Biotechnology (Japan) and Roche (Indianapolis, IN), respectively. Rabbit anti‐rat α‐SMA antibody, rabbit anti‐rat collagen antibody, rabbit anti‐rat TIMP1 antibody, and horseradish peroxidase (HRP)‐conjugated goat anti‐rabbit antibody were purchased from Abcam (Abcam, UK).

*Preparation of Nanoplexes*: The non‐VA‐decorated copolymer PEG–bPEI–PAsp(DIP–BzA), abbreviated as PBP, was synthesized according to the recent report.^60^ The synthesis of VA‐terminated copolymer VA–PEG–bPEI–PAsp(DIP–BzA), abbreviated as T‐PBP, is described in Figure S1 in the Supporting Information. Preparation of SPIO‐loaded micelles (i.e., PBP/S and T‐PBP/S) and SPIO‐free micelles (i.e., PBP and T‐PBP) is outlined in Figure 1. Briefly, polymer (25 mg) with or without mixing with SPIO (2.5 mg) was dissolved in a mixture of CHCl_3_ (10 mL) and MeOH (2 mL), and then emulsified in 25 mL of deionized water under sonication (60 Sonic Dismembrator, Fisher Scientific). The solution was rotoevaporated to remove CHCl_3_ and then dialyzed (molecular weight cutoff = 14 kDa) against water for 1 d. The dialyzed solution was then filtered through a 220 nm syringe filter to remove large aggregates. Finally, the solution was stored at 4 °C prior to further experiments.

The above micelle solution was diluted to different concentrations with Tris–HCl buffer (pH 7.4). A certain amount of micelle solution was mixed with a predetermined amount of miRNA in Tris–HCl buffer (pH 7.4). The mixture was gently pipetted for 5 min and then kept still at room temperature for 30 min to allow formation of nanoplex. By this approach, a series of nanoplexes including N‐SCR (PBP micelle complexing scrambled miRNA), N‐SCR/S (PBP micelle complexing scrambled miRNA and encapsulating SPIO), T‐SCR (T‐PBP micelle complexing scrambled miRNA), and T‐SCR/S (T‐PBP micelle complexing scrambled miRNA and encapsulating SPIO) at different N/P ratios were formed. N/P ratio was calculated as the molar number of nitrogen atoms in the PEI block over that of the phosphate groups in the miRNA.

*Characterizations*: ^1^H NMR spectra were recorded on a Bruker 400 MHz spectrometer at room temperature. Gel permeation chromatography (Breeze, Waters) was performed to evaluate the molecular weights and molecular weight distributions of copolymers using a differential refractive index detector. The 1 g L^−1^ LiBr‐containing dimethylformamide was used as mobile phase at a flow rate of 1 mL min^−1^, and polystyrene standards (Sigma) were used for column calibration. Particle size and zeta potential were measured by dynamic light scattering at 25 °C (Malvern NANO ZS, UK). TEM analyses were performed using JEM 1400 Plus operated at 120 kV (JEOL, Japan). Samples on copper grid were stained with uranyl acetate (0.3% w/v) if required.

*Agarose Gel Electrophoresis*: The nanoplexes N‐SCR, T‐SCR, and T‐SCR/S with various N/P ratios (0, 2, 5, 10, 15, 20, and 25) at pH 7.4 were electrophoresed in 1% agarose gel containing EB (0.5 µg mL^−1^). The electrophoresis was conducted for 15 min under 120 V in a TAE buffer solution (40 × 10^−3^ m Tris–HCl, 1% v/v acetic acid, and 1 × 10^−3^ m ethylenediaminetetraacetic acid). The staining of miRNA was attributed to the binding of EB to short intrastrand helical regions in the molecule. The images of EB‐stained miRNA were acquired with a DNR Bio‐Imaging System (DNR Bio‐Imaging Systems Ltd., Israel).

*Cell Culture*: Immortalized rat HSC cell line (HSC‐T6) was cultured under 5% CO_2_ in DMEM (Gibco) supplemented with 10% fetal bovine serum at 37 °C. When their confluence reached 80–90%, the cells were trypsinized and subcultured.

*Cell Viability Assay*: HSCs were seeded in a 96‐well plate at a density of 5 × 10^3^ per well in DMEM containing 10% FBS in a humidified atmosphere of 5% CO_2_. After being incubated for 24 h at 37 °C, the cells were treated with N‐SCR, T‐SCR, and T‐SCR/S. The culture medium in each well was replaced with 100 µL of fresh medium, and various nanoplexes (N/P 10) at different concentrations were added. After 24 h incubation, the cell viabilities were measured by MTT assay. Briefly, 20 µL of MTT solution was added to each plate well. The cells were incubated at 37 °C for 2 h. The absorbance at 562 nm was measured using a microplate reader (Tecan, Germany). The dose of SCR was 100 × 10^−9^ m in each well. The cytotoxicities of N/SCR and T/SCR at various N/P ratios were investigated as well. Cell viability assay was performed in triplicate for all tests.

*Intracellular Distribution of Nanoplexes*: The intracellular distribution of Cy3‐labeled SCR was detected using confocal laser scanning microscopy (CLSM). HSCs were seeded in a confocal dish at a density of 5 × 10^3^ cells per dish and incubated in 1 mL of DMEM supplemented with 10% FBS overnight. Hoechst 33342 was added and incubated for 15 min to stain nuclei. Then, the T‐SCR nanoplex (N/P 10) at a SCR concentration of 100 × 10^−9^ m was added. At different time points, the cells were incubated with LysoTracker Green DND‐26 to stain lysosomes, washed with PBS three times, and then imaged using CLSM.

*In Vitro miRNA Transfection*: To evaluate the RBP receptor–mediated transportation of T‐SCR into cells, HSCs were incubated in the presence of RBP (0.7 µg mL^−1^). For the free ligand competitive inhibition assay, HSCs were pretreated with excessive free vitamin A (0.5 µg mL^−1^) for 2 h before incubation with Cy3‐labeled T‐SCR. Then, the cells were washed three times with PBS and fixed with 4% paraformaldehyde for 10 min at room temperature. Afterward, the cells were washed three times with PBS and stained with DAPI for 2 min to show nuclei. Finally, after washing with PBS three times, the fluorescence of SCR and nuclei was observed under a CLSM (Nikon C2, Japan). Cy3 and DAPI were excited at 514 and 358 nm, respectively.

Flow cytometry assay was performed to quantitatively determine transfection efficiency. To evaluate the effect of N/P ratios, cells were incubated with Cy3‐labeled T‐SCR nanoplexes of different N/P ratios (2, 5, 10, 15, and 20). Then, cells were collected and analyzed with flow cytometry (CytoFlex S, Beckman Coulter, USA). To evaluate RBP‐mediated cellular uptake, Cy3‐labeled N‐SCR and T‐SCR nanoplexes were formed at N/P 10. HSCs were treated with N‐SCR, T‐SCR, T‐SCR plus RBP (0.7 µg mL^−1^), and T‐SCR plus preincubation with free vitamin A (0.5 µg mL^−1^) in PBS solution for 0.5 h or in serum‐containing culture medium for 2 h. Cells were washed with PBS three times, trypsinized, resuspended in 500 µL of ice‐cold PBS, and analyzed with flow cytometry (CytoFlex S, Beckman Coulter, USA). The dose of Cy3‐labeled SCR was 100 × 10^−9^ m per well.

*Prussian Blue Staining*: HSCs were seeded in a six‐well plate at 5 × 10^4^ cells per well in a humidified atmosphere of 5% CO_2_. When their confluence reached 90%, cells were incubated with SPIO‐encapsulated N‐SCR (N‐SCR/S) and SPIO‐encapsulated T‐SCR (T‐SCR/S) (N/P 10) for 2 h. After washing three times with PBS, cells were treated with Prussian blue solution containing 2% hydrochloride and 2% potassium ferrocyanide(II) trihydrate for 30 min at 37 °C. Then, cells were washed three times with PBS and observed under a microscope.

*In Vitro and In Vivo MRI Scanning*: HSCs seeded in a six‐well plate at 1 × 10^5^ cells per well were incubated with N‐SCR/S and T‐SCR/S at various Fe concentrations of 0, 5, 10, 20, and 40 µg mL^−1^ in serum‐containing DMEM medium for 2 h. After washing three times with PBS, the cells were trypsinized and then resuspended in a 1% gelatin solution. The MR signals were monitored with a 3.0 T MR scanner (GE Healthcare, UK). In animal study, the CCl_4_‐induced liver fibrotic rats were randomly separated into two groups and injected with N‐SCR/S nanoplex and T‐SCR/S nanoplex, respectively. The normal rats were i.v. injected with T‐SCR/S nanoplex as control. In vivo magnetic resonance imaging was performed on a clinical 1.5 T system (Intera, Philips Medical Systems, Netherlands) with an animal coil specifically for the rat imaging. The detailed scanning procedures are described in the Supporting Information.

*Quantitative Real‐Time PCR and Western Blot Assays*: Relative mRNA and protein expression levels of COL1A1, α‐SMA, and TIMP1 in HSCs or in liver of rats after different nanoplex treatments were evaluated via quantitative real‐time PCR and Western blot assays. The contents of mature miRNA‐29b and miRNA‐122 in rats receiving various nanoplex treatments were measured by real‐time PCR as well. The detailed experiments are described in the Supporting Information.

*Animal Models of Liver Fibrosis*: Male Sprague‐Dawley rats (180–200 g) were purchased from Sun Yat‐sen University (Guangzhou, China). All animal experiments were performed in accordance with Guide for the Care and Use of Laboratory Animals and approved by the Institutional Animal Care and Use Committee of the Sun Yat‐sen University. Liver fibrosis was induced by twice‐a‐week intraperitoneal (i.p.) injection of CCl_4_ and olive oil mixture (1:1 in volume ratio) at 2 mL kg^−1^ body weight per injection.^68^ Control animals were i.p. administered with only olive oil. After the rats were anesthetized with sodium pentobarbital (40 mg kg^−1^ body weight, intraperitoneally), liver and serum samples were obtained and subjected to pathological and functional analyses.

*Colocalization of miRNA and Activated HSCs in Fibrotic Liver*: Immunofluorescence staining was performed to reveal the localization of miRNA in the fibrotic liver sections. Nanocarrier was labeled with Rho, emitting red fluorescence under confocal laser microscopy. The α‐SMA in activated HSCs was immunostained with AF488‐conjugated antibody (green fluorescence). The detailed experiments are described in the Supporting Information.

*Targeted Delivery of miRNA in Liver Fibrosis Rats*: To evaluate the HSC‐targeted delivery efficiency of miRNA, targeting (T‐PBP) and nontargeting (PBP) micelles complexing various miRNAs were administered via tail vein at a miRNA dose of 1 mg kg^−1^ body weight. Then, liver tissues of rats were harvested at 1, 2, or 3 d after i.v. administration. Real‐time PCR was used to determine the relative expression levels of miRNA‐29b and miRNA‐122 in fibrotic livers.

*In Vivo miRNA‐29b and miRNA‐122 Treatment*: To evaluate the therapeutic effects of various nanoplexes in liver fibrotic rats, seven groups (*n* = 8) of rats were treated with different nanoplexes. One group receiving i.p. injection of olive oil was used as control; the other six groups receiving i.p. injection of the CCl_4_ and oil mixture were treated with PBS, T‐SCR (T‐PBP micelle complexing scrambled miRNA), N‐Mix (PBP micelle complexing miRNA‐29b and miRNA‐122), T‐29b (T‐PBP micelle complexing miRNA‐29b), T‐122 (T‐PBP micelle complexing miRNA‐122), and T‐Mix (T‐PBP micelle complexing miRNA‐29b and miRNA‐122), respectively, 1 d after the injection of CCl_4_ and oil mixture. The dose of miRNA was 1 mg kg^−1^ body weight if needed, and the formulations were administered via tail vein at 1 mL kg^−1^ body weight. The survival rates of rats in all groups remained above 90% during 6 weeks. After rats were sacrificed under anesthesia at 6 weeks posttreatment, the liver tissues and blood samples were harvested for histopathological studies. The levels of serum biochemical markers for liver injury, such as ALT, AST, and T‐BIL levels, were measured by standard procedures following the manufacturer's instructions.

*Immunohistochemistry*: Liver tissue sections were fixed in 10% paraformaldehyde overnight and then embedded in paraffin. The 5 µm thick paraffin‐embedded sections were subjected to H&E staining and Sirius red staining according to the manufacturer's instructions. The sections were observed under an Olympus BX51 microscope (Olympus Co., Japan).

*Statistical Analysis*: Statistical analysis was performed using an analysis of variance with Graph Prism 6.0. The results were shown as mean ± standard deviation (SD), and *P < 0.05 was considered statistically significant.

## Conflict of Interest

The authors declare no conflict of interest.

## Supporting information

Supplementary
