# Supplementary material for: Synergistic MicroRNA Therapy in Liver Fibrotic Rat Using MRI‐Visible Nanocarrier Targeting Hepatic Stellate Cells
Source: Adv Sci (Weinh). 2019 Jan 15;6(5):1801809. doi: 10.1002/advs.201801809 (PMC6402399; doi:10.1002/advs.201801809)
Supplement: Supplementary file 1 — Supplementary [file ADVS-6-1801809-s001.pdf]

Copyright WILEY-VCH Verlag GmbH & Co. KGaA, 69469 Weinheim, Germany, 2018.

ADVANCED SCIENCE
Open Access

Supporting Information

for *Adv. Sci.,* DOI: 10.1002/advs.201801809

Synergistic MicroRNA Therapy in Liver Fibrotic Rat Using MRI-Visible Nanocarrier Targeting Hepatic Stellate Cells

Jun Wu, Jinsheng Huang, Sichi Kuang, Jingbiao Chen, Xiaoxia Li, Bin Chen, Jin Wang, Du Cheng,* and Xintao Shuai**

Supporting Information

Synergistic microRNA therapy in liver fibrotic rat using MR-visible nanocarrier targeting hepatic stellate cells

Jun Wu, Jinsheng Huang, Sichi Kuang, Jingbiao Chen, Xiaoxia Li, Bin Chen, Jin Wang, Du Cheng, and Xintao Shuai

Synthesis of VA-PEG-bPEI-PAsp(DIP-BzA) Copolymer

The polymer $bPEI_{1.8k}$-PAsp(DIP-BzA) was synthesized according to our recent report,[1] and the grafting densities of DIP and BzA were designed to be 30 and 10, respectively. The VA-PEG-bPEI-PAsp(DIP-BzA) (T-PBP) was synthesized by a reaction between VA-PEG-CDI and bPEI-PAsp(DIP-BzA). After vitamin A (VA, 0.25 g) was dissolved in 3 mL of anhydrous THF, N, N′-Carbonyldiimidazole (CDI, 0.28 g) was added dropwise.[2] The mixture solution was stirred for 10 h at room temperature. Then, NH_2-PEG-OH (0.6 g, *M*n*:* 2.3 k) was added into the above mixture solution. The solution was continuously stirred for 10 h at room temperature. After completion of reaction, the mixture was precipitated into 100 mL of ether, frozen, filtrated, washed with ether and then vacuum-dried to obtain activated VA-PEG-OH (Yield: 85%). Next, VA-PEG-OH (0.5 g) was dissolved in 5 mL of anhydrous THF, followed by adding CDI (97 mg) and then the solution was stirred for 12 h at room temperature. Upon completed reaction, the mixture

was precipitated into 100 mL of ether, frozen, filtrated, washed with ether and then vacuum-dried to obtain activated VA-PEG-CDI (Yield: 90%). Finally, bPEI-PAsp(DIP-BzA) (0.56g, *M*n*:* 11.2k) was dissolved in 3 mL of DMSO, followed by addition of VA-PEG-CDI (0.26 g). The solution was stirred for 0.5 h at room temperature, dialyzed against methanol for 2 d, rotary evaporated and then vacuum-dried to obtain the VA-decorated triblock copolymer (Yield: 81%). The non-targeting copolymer poly(ethyleneglycol)-polyethyleneimine-poly(N-(N',N'-diisopropylaminoethyl)-*co*-benzylamino)aspartamide, abbreviated as PEG-bPEI-PAsp(DIP-BzA) or PBP, was also synthesized using the same above procedures.

In vitro and in vivo MRI Scanning

HSCs seeded in a 6-well plate at 1×10^5 cells per well were incubated with N-SCR/S and T-SCR/S at various Fe concentrations of 0, 5, 10, 20 and 40 μg/mL in DMEM medium for 2 h. After washing three times with PBS, the cells were trypsinized and then resuspended in a 1% gelatin solution. The MR signals were monitored with a 3.0-T MR scanner (GE Healthcare, UK). A head coil with an inner diameter of 8 inches was used for the cell imaging. The T_2-weighted images were acquired using the following parameters: repetition time (TR)/echo time (TE), 2000/100 ms; field of view (FOV), 120 mm; matrix, 256×256; slice thickness, 1 mm. The signal intensities of N-SCR/S- or T-SCR/S-treated

cells were determined using a circular 10 mm^2 region of interest. The relative signal intensity of treated cells was normalized using control cells without nanoplex treatment.

In animal study, the CCl_4-induced liver fibrotic rats were randomly separated into two groups and injected with N-SCR/S and T-SCR/S nanoplexes, respectively. The normal rats were i.v. injected with T-SCR/S nanoplex as control. In vivo magnetic resonance imaging was performed on a clinical 1.5-T system (Intera, Philips Medical Systems, Netherlands) with an animal coil specifically for the rats imaging. T_2-weighted images were acquired using the following parameters: TR/TE, 1836/100 ms; FOV, 70×60×28 mm; matrix, 232 × 258; slice thickness, 1.5 mm; relative signal noise ratio (Rel. SNR). The normalized MR signal intensities were calculated by the mean T2 signal intensities of three largest transverse sections of liver tissue as compared with those of the muscle in the same section.

Quantitative Real-Time PCR

To evaluate the mRNA expressions of COL1A1, α-SMA and TIMP1, total RNA was extracted from cell pellets or liver tissues using Trizol reagent (Invitrogen, USA) according to the manufacturer's protocol. Total RNA (1 μg) were used for reverse transcription in total volume of 20 μL using PrimeScript™ RT Master Mix (Takara Biotechnology, Japan) on a Thermal Cycler (Applied Biosystems, USA). Primer sequences used for the PCR experiment were shown in Table S1. Quantitative real-time

PCR was performed using FastStart Universal SYBR Green Master (ROX) (Roche, Indianapolis, IN) on a StepOne Plus real-time PCR System (Applied Biosystems, USA) with β-actin as an internal control.

To evaluate the miRNA levels of miRNA-29b and miRNA-122, total RNA was first reverse transcribed into cDNA using SYBR Green microRNA assay (Takara Biotechnology, Japan). The comparative ΔCt method was used to calculate the relative levels of mature miRNA-29b and miRNA-122 with RNU6 as an endogenous control (Fold difference relative to RNU6). The PCR reaction was performed as follows: 95 ^{o}C for 10 min, and 40 cycles of 95°C for 15 s, 60 ^{o}C for 60 s and 95°C for 15 s.

Table S1. **Primer sequences for quantitative real-time PCR**

Genes	Sequences
β-actin	5’-GGAGATTACTGCCCTGGCTCCTA-3’
	5’-GACTCATCGTACTCCTGCTTGCTG-3’
COL1A1	5’-CTGACTGGAAGAGCGGAGAG-3’
	5’-GAGTGGGGAACACACAGGTC-3’
α-SMA	5’-CAGGGAGTGATGGTTGGAAT-3’
	5’-GGTGATGATGCCGTGTTCTA-3’
TIMP1	5’-TGCAACTCGGACCTGGTTAT-3’
	5’-ACAGCGTCGAATCCTTTGAG-3’
miR-29b	5’-CGCGCGTTTCATATGGTGGTTTAGATTT-3’
miR-122	5’- ACGTGGAGTGTGACAATGGTGTTTG-3’

Western Blot Assay

Total protein was collected from cell pellets or liver specimens (10 mg), and the protein concentration was measured using a bicinchoninic acid protein assay kit (Shenggong Bio-Tech., China). These samples were boiled for 5 min before gel loading. About 30 μg of protein of each sample were separated on a 12% SDS-PAGE in the tank (Bio-rad, CA), transferred to polyvinylidene difluoride (PVDF) membranes (Life Technology) and then blocked with 5% BSA for 1 h at room temperature. These membranes were incubated with rabbit anti-rat α-SMA antibody (dilution 1:500 in TBST), rabbit anti-rat COL1A1 antibody (dilution 1:1000 in TBST), and rabbit anti-rat TIMP1 antibody (dilution 1:1000 in TBST) at 4°C overnight if required, and then incubated with horseradish peroxidase (HRP)-conjugated goat anti-rabbit antibody (dilution 1:2000 in TBST) for 1 h at room temperature. Lastly, the protein bands were visualized via a chemi-luminescence system (Amersham Biosciences, USA) with ECL detection reagents. GAPDH was served as an internal standard to normalize protein expression.

Colocalization of miRNA and Activated HSCs in Fibrotic Liver

Immunofluorescence staining was performed to reveal the localization of miRNA in the fibrotic liver sections. Nanocarrier was labeled with Rhodamine B (Rho), emitting red fluorescence under confocal laser microscopy. The α-SMA in activated HSCs was

immunostained with Alexa Fluor 488 (AF488)-conjugated antibody (green fluorescence). One day after the last administration of CCl_4 mixture, the fibrotic rats were intravenously (i.v.) injected with Rho-labeled N-SCR or Rho-labeled T-SCR nanoplexes. At 2 h after i.v. injection, the livers were collected and fixed in 10% PBS buffered formalin for 24 h. The fixed samples were exposed to 10% and 30% sucrose in PBS for 12 h in sequence. The samples were then embedded in Tissue Tek OTC compound (Sakura Finetek, CA) and frozen at -25 °C. For immunological detection, 5 μm thick frozen sections were fixed with the precooled acetone for 10 min and rinsed 4 times in PBS. The sections were blocked with 5% BSA for 1 h at room temperature to avoid nonspecific binding. Next, the sections were incubated with a primary antibody (dilution 1:200) against α-SMA at 4°C overnight, washed with TBST three times and then incubated with an AF488-conjugated secondary IgG (dilution 1:200) for 1.5 h at room temperature. Nuclei were counterstained by DAPI for 5 min. Images were captured by confocal microscopy.

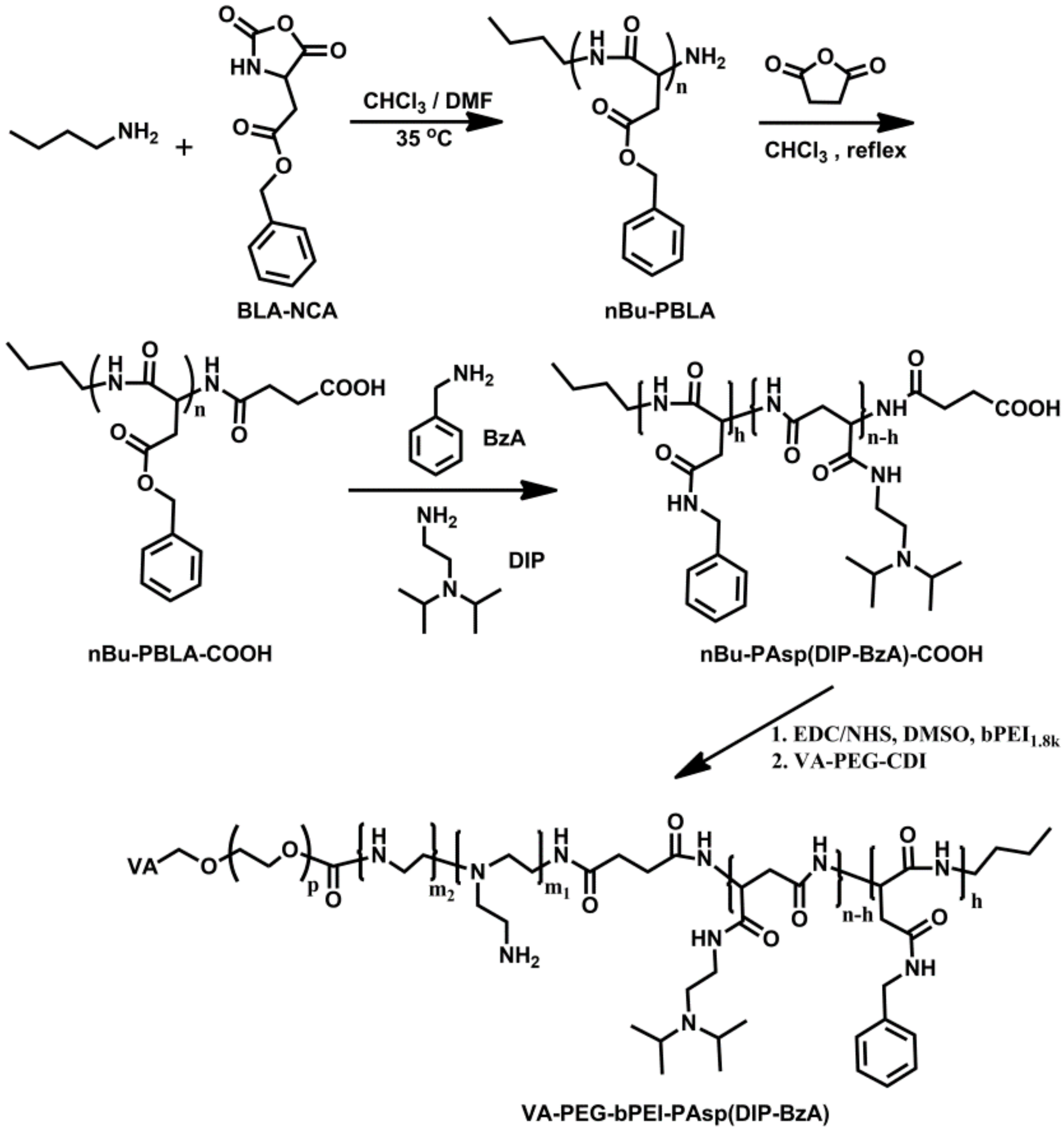

Figure S1. **Synthetic route of the vitamin A-terminated triblock copolymer VA-PEG-bPEI-PAsp(DIP-BzA) denoted as T-PBP.**

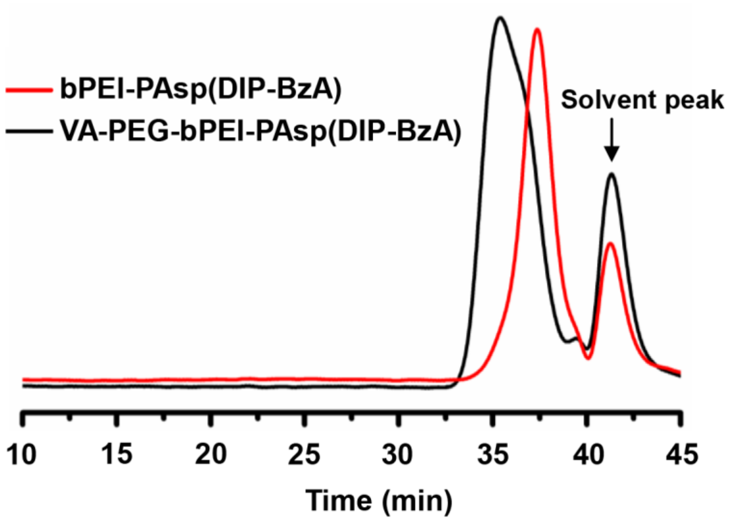

Figure S2. GPC curves of bPEI-PAsp(DIP-BzA) and VA-PEG-bPEI-PAsp(DIP-BzA) in DMF containing LiBr (1 g/L) at a flow rate of 1 mL/min.

Table S2. Characteristics of the synthesized polymers

Polymer	M_n^a (kDa)	M_n^b (kDa)	M_w/M_n^b
bPEI-PAsp(DIP-BzA)	11.2	9.3	1.20
VA-PEG-bPEI-PAsp(DIP-BzA)	13.7	12.5	1.33

[a]calculated by ^{1}H NMR; [b]calculated by GPC.

Table S3. Averaged particle size and zeta potential of nanoplexes (N/P=10) in PBS solution at different pH. Results are presented as mean ± SD (n = 3)[a]

**Nanoplex**	**pH**	**Size / nm**	**PDI**[b]	**ζ potential / mV**
**T-SCR/S**	7.4	168.8 ± 9.2	0.11	+12.12 ± 3.59
	5.0	1088 ± 108.5	0.77	+9.50 ± 0.75
**N-SCR/S**	7.4	166.2 ± 6.4	0.10	+11.90 ± 3.32
	5.0	1306 ± 82.0	0.46	+9.27 ± 1.29

[a]All data were measured by dynamic light scattering (DLS). [b]PDI means particle distribution index.

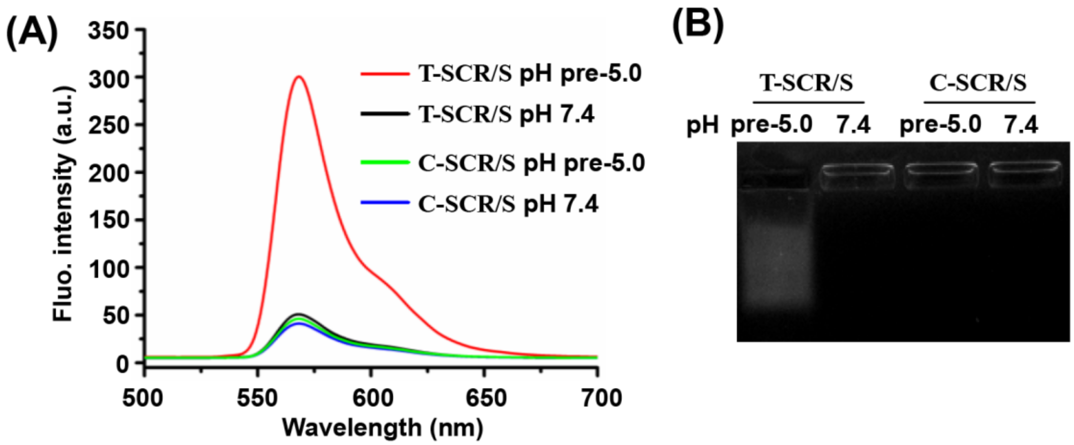

Figure S3. In vitro release profile of scrambled miRNA (SCR) from T-SCR/S and C-SCR/S nanoplexes affected by preincubation at different pH. (**A**) Fluorescence intensity of Cy3-labeled SCR complexed with T-SCR/S and C-SCR/S. SCR was labeled with fluorescent dye Cy3 for monitoring miRNA release. Cy3-labeled SCR was 0.5 μM. (**B**) Electrophoretic mobility of T-SCR/S and C-SCR/S in agarose gel (pH 7.4) after treated with buffered solution of pH 5.0 and pH 7.4. The control nanoplex (C-SCR/S) was prepared by using SPIO-loaded cationic micelle of VA-PEG-bPEI-PAsp(BzA) without DIP grafting to complex SCR. The T-SCR/S and C-SCR/S nanoplexes were both prepared at NP 10. The measurement of fluorescent intensity and gel electrophoresis was performed at pH 7.4 no matter whether nanoplex was preincubated at pH 5.0 or not. The group of pH pre-5.0 indicated that the nanoplex was preincubated at pH 5.0.

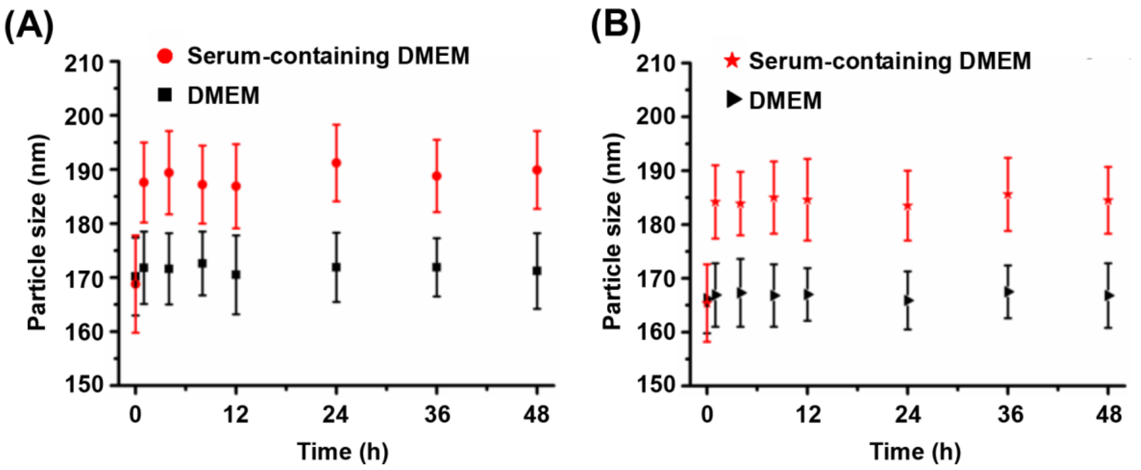

Figure S4. **Stability of nanoplexes**. (**A**) T-SCR/S and (**B**) N-SCR/S in Dulbecco's modified eagle medium (DMEM, pH 7.4) and 10% fetal bovine serum (FBS)-containing DMEM (pH 7.4) as measured by dynamic light scattering (mean ± SD, n =3). The T-SCR/S and N-SCR/S nanoplexes kept stable in DMEM. Moreover, the particle size of these two nanoplexes only showed a slight increase and then kept stable over 48 h in serum-containing DMEM.

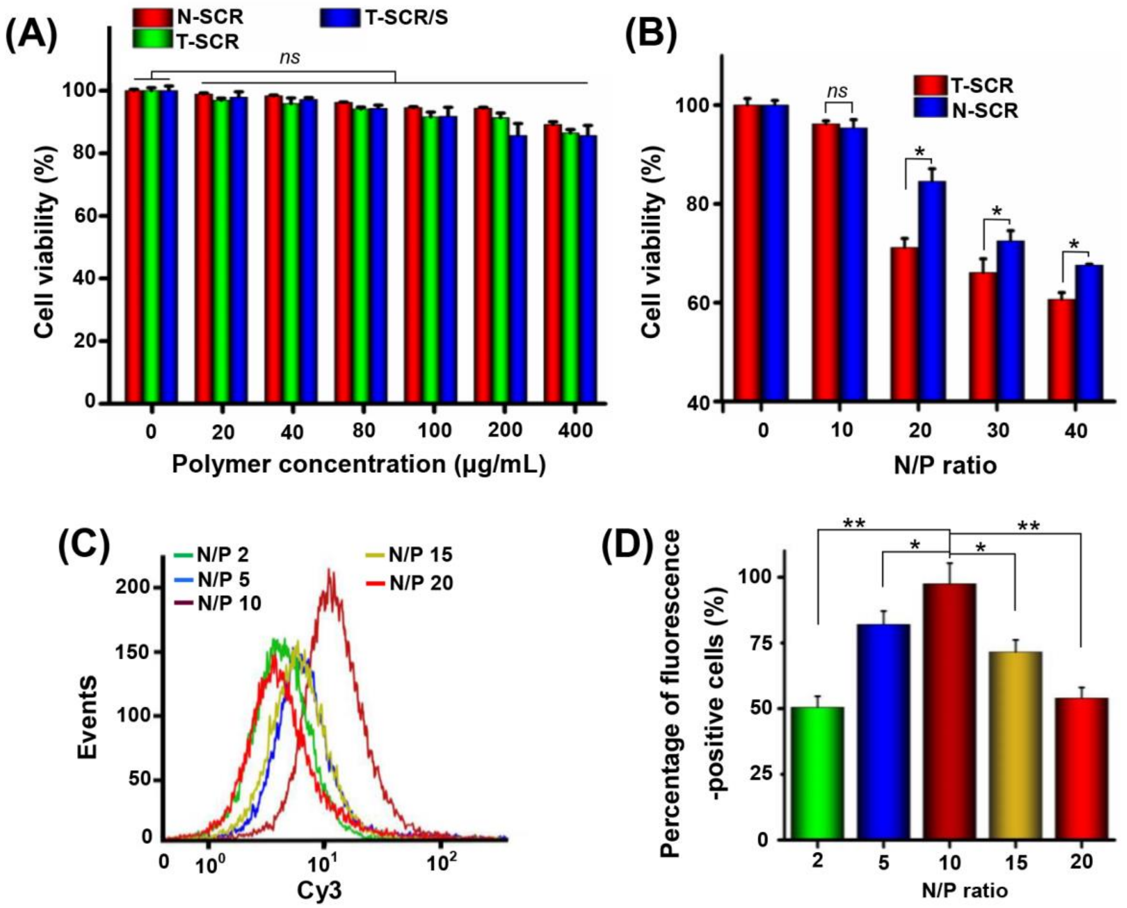

Figure S5. **Cytotoxicity of SCR-complexed nanoplexes and effect of N/P ratios on gene transfection efficiency.** (**A**) Viabilities of HSCs incubated with different concentrations of non-targeting nanoplex (N-SCR), targeting nanoplex (T-SCR), and targeting nanoplex loaded with SPIO (T-SCR/S). Nanoplexes were prepared at an N/P ratio of 10. (**B**) Viabilities of HSCs incubated with 100 μg/mL T-SCR nanoplex and 100 μg/mL N-SCR nanoplex at different N/P ratios. (**C**) Effect of N/P ratios on the transfection efficiency of T-SCR nanoplex as determined by flow cytometry. (**D**) Statistic data of miRNA transfection efficiency from (C). Incubation times were 24 h for (A-B) and 2 h for (C-D), respectively. Data are shown as the mean ± SD, n = 3. *$P < 0.05$, **$P < 0.01$ and ns: no significant difference. Serum-containing DMEM was used to culture cells.

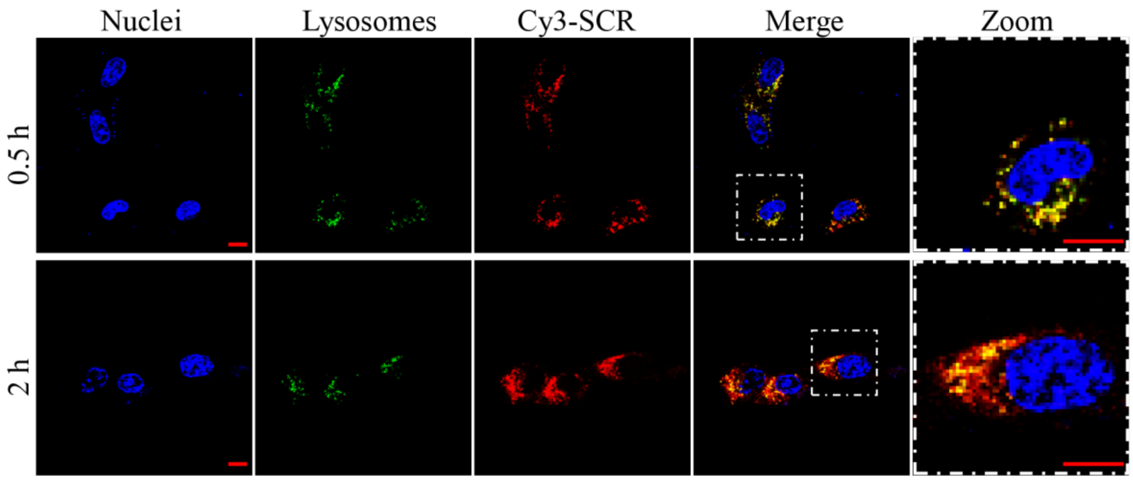

Figure S6. **Intracellular distribution of Cy3-labelled T-SCR at different time points after cell transfection.** Cy3-SCR was Cy3 fluorescent molecule-labelled SCR miRNA (red fluorescence) and complexed with T-PBP micelle at N/P 10. The nuclei and lysosomes were stained blue and green with Hoechst 33342 and LysoTracker® Green DND, respectively. The scale bars represent 25 μm.

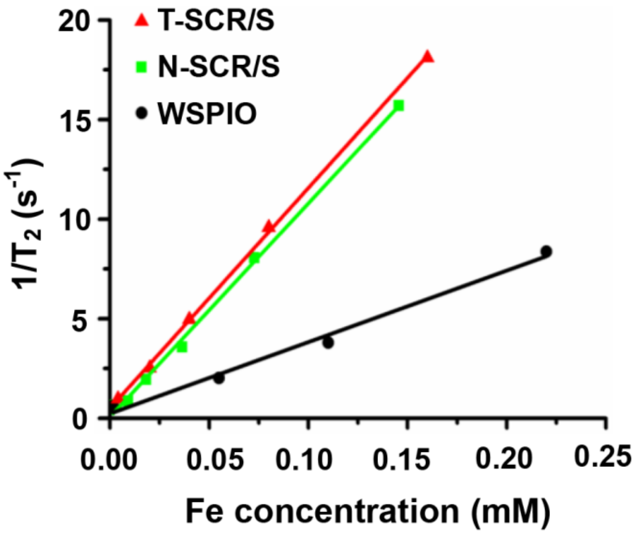

Figure S7. **Magnetic resonance T_2 relaxation rate of WSPIO, N-SCR/S and T-SCR/S.** The T_2 relaxivity (r_2) of T-SCR/S and N-SCR/S were 110.6 and 107.2 Fe $mM^{-1}s^{-1}$, respectively, which were significantly higher than that of WSPIO (r_2=35.9 Fe $mM^{-1}s^{-1}$). Abbreviations: T-SCR/S, T-PBP micelle complexing SCR and encapsulating SPIO; N-SCR/S, PBP micelle complexing SCR and encapsulating SPIO; WSPIO, Water soluble single SPIO nanoparticle.

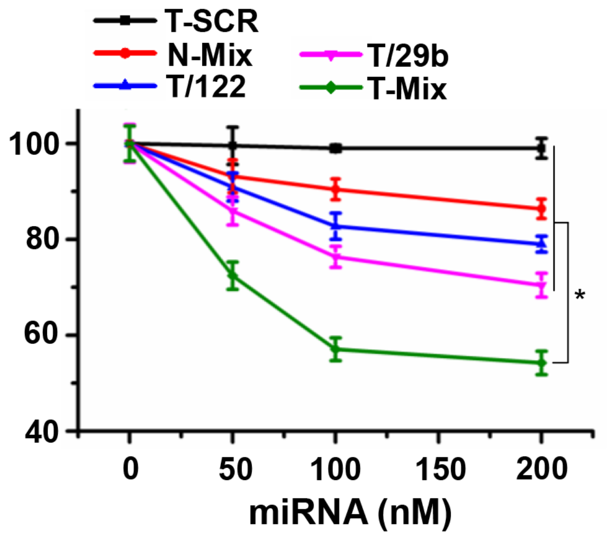

Figure S8. **Viabilities of HSCs incubated with different nanoplexes.** Nanoplexes were prepared at an N/P ratio of 10. Incubation time was 72 h. Data are shown as the mean ± SD, n=3. **P*<0.05.

Abbreviations: T-SCR, T-PBP micelle complexing SCR; N-Mix, PBP micelle complexing miRNA-29b and miRNA-122; T-122, T-PBP micelle complexing miRNA-122; T-29b, T-PBP micelle complexing miRNA-29b; T-Mix, T-PBP micelle complexing miRNA-29b and miRNA-122.

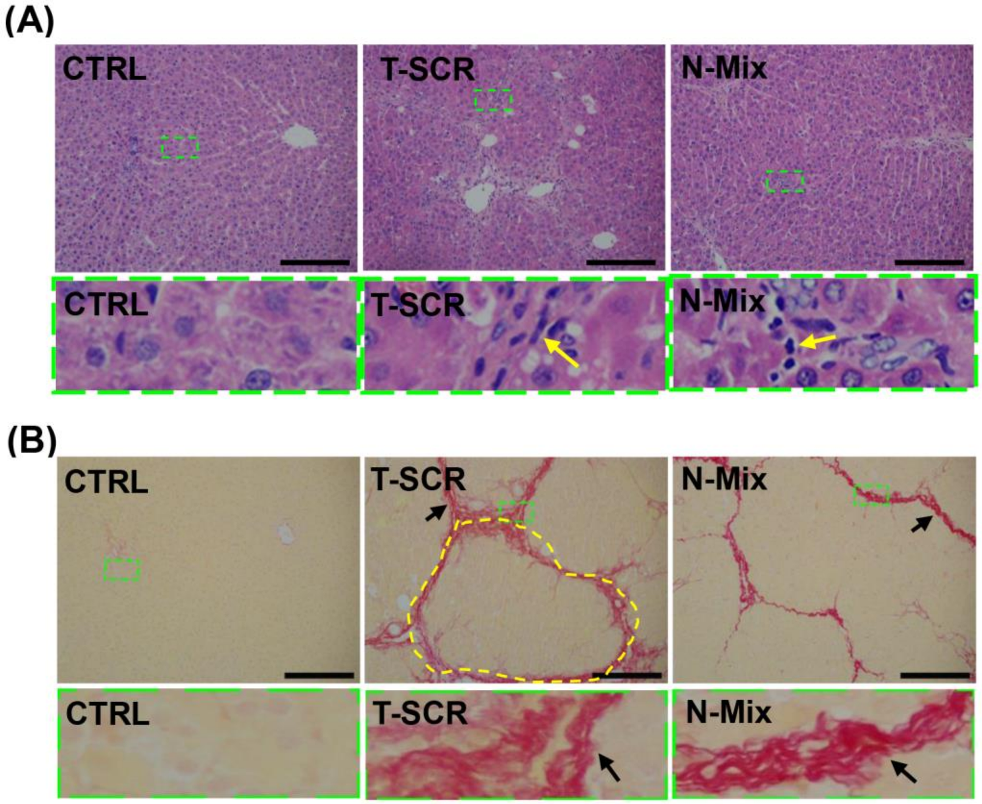

Figure S9. **In vivo anti-fibrotic effect of miRNA treatments.** (**A**) Hematoxylin & eosin (H&E) and (**B**) Sirius red staining of liver specimen of CCl_4-induced rats after treatment of various nanoplexes. The areas marked with dotted green rectangle are enlarged to reveal the changes of pathological structure. The yellow arrows indicate the inflammatory cells. The black arrows mark the collagen fiber. The yellow dotted portion indicates the pseudolobule in liver. Scale bars represent 100 μm.

Abbreviations: CTRL, normal rats treated with equal quantity of olive oil; T-SCR, CCl_4-induced liver fibrotic rats treated with T-PBP micelle complexing SCR; N-Mix, CCl_4-induced liver fibrotic rats treated with PBP micelle complexing miRNA-29b and miRNA-122.

References

[1] J. Huang, C. Lin, J. Fang, X. Li, J. Wang, S. Deng, S. Zhang, W. Su, X. Feng, B. Chen,

D. Cheng, X. Shuai, *ACS Appl. Mater. Interfaces.* **2018**, 10, 28471.

[2] Y. Sato, K. Murase, J. Kato, M. Kobune, T. Sato, Y. Kawano, R. Takimoto, K. Takada, K. Miyanishi, T. Matsunaga, T. Takayama, Y. Niitsu, *Nat. Biotechnol.* **2008**, 26, 431.
